# Clinical and epidemiological characterization of heart failure patients in a high-altitude setting: a retrospective study at a tertiary hospital in Quito, Ecuador

**DOI:** 10.3389/fcvm.2026.1776502

**Published:** 2026-04-21

**Authors:** Vladimir Ullauri, Marlon Patricio Aguirre Espinosa, Tanya María Padilla Molina, Diana Moreira-Vera, Henry Oswaldo Jaramillo Prado, Ana Gabriela Finke Barriga, John Fabricio Salto González, Liliana Elizabeth Flores Rodríguez, Marisol Elizabeth Cárdenas Calderón, Giuseppe Paul Ayala Abarca, Juan S. Izquierdo-Condoy, Jorge Vasconez-Gonzalez, Esteban Ortiz-Prado

**Affiliations:** 1 Departamento de Cardiología, Hospital Metropolitano de Quito, Quito, Ecuador; 2 One Health Research Group, Universidad de las Americas, Quito, Ecuador

**Keywords:** cardiovascular diseases, Ecuador, epidemiology, heart failure, high altitude

## Abstract

**Background:**

Heart failure (HF) is a major global health problem and a leading cause of morbidity and mortality. In Latin America, evidence remains limited, and HF characterization in high-altitude care settings is underreported. Quito (−2,800 m a.s.l.) provides a unique clinical context, although retrospective hospital-based data without standardized hypoxia phenotyping or sea-level comparators cannot support altitude-specific causal inference.

**Objective:**

This study aims to describe the clinical and epidemiological characteristics of patients diagnosed with HF at the Metropolitan Hospital of Quito, a tertiary care facility located at an altitude of approximately 2,800 meters, from January 2021 to December 2023.

**Methods:**

A retrospective observational study was conducted using anonymized medical records of 122 patients diagnosed with HF (ICD-10 codes I500, I501, I509). Data on demographic, clinical, and outcome variables were collected. Exploratory comparisons were performed by discharge survival status (alive vs. deceased) using chi-square or Fisher's exact tests for categorical variables and two-sample Student's *t*-tests for continuous variables (two-sided *p* < 0.05).

**Results:**

Most patients (88.5%) were aged over 65 years, with men comprising 55.7% of the cohort. Hypertension (59.8%), dyslipidemia (18.9%), and atrial fibrillation (44.3%) were the most prevalent comorbidities. Hypertensive heart disease was the most frequent documented etiology of HF (14.8%), although etiology was unavailable in a substantial proportion of records. In-hospital mortality was low (3.3%). Exploratory univariate analyses identified unadjusted associations between in-hospital mortality and dialysis dependency, immunologic diseases, and other vascular diseases.

**Conclusions:**

This study provides a contemporary clinical and epidemiological profile of HF patients managed at a high-altitude tertiary hospital in Quito and identifies exploratory factors associated with in-hospital mortality in this care setting. Future multicenter studies incorporating standardized hypoxia-related measurements and appropriate comparator cohorts are needed to better understand HF phenotypes and outcomes in Andean populations.

## 1 Introduction

The heart functions as a physiological pump responsible for maintaining a cardiac output of approximately 5 to 6 L per minute to meet the metabolic demands of the body (1). Heart failure (HF) arises when the heart is unable to adequately perform this function due to intrinsic or extrinsic factors (2). When present, HF is associated with a complex clinical syndrome resulting from structural or functional cardiac abnormalities that impair ventricular performance (3). These abnormalities can manifest as reduced ventricular filling (diastolic dysfunction) or diminished blood ejection (systolic dysfunction), ultimately leading to hemodynamic instability and inadequate tissue perfusion (4, 5). While left ventricular dysfunction is the most common underlying cause, heart failure can also arise from abnormalities involving the pericardium, endocardium, heart valves, or great vessels (6). HF may manifest acutely or progress gradually into a chronic condition. Key etiologies include coronary artery disease, hypertension, cardiomyopathies, cardiotoxic agents, and inflammatory or infiltrative cardiac disorders (4, 7).

Heart failure is classified using several frameworks, with the American College of Cardiology and American Heart Association (ACC/AHA) staging system being the most widely used (8). This system categorizes HF into four progressive stages, from individuals at risk without structural heart disease (Stage A) to advanced disease with severe symptoms affecting daily life (Stage D) (9). Additionally, HF is clinically divided into heart failure with preserved ejection fraction (HFpEF) or reduced ejection fraction (HFrEF) (10). The New York Heart Association (NYHA) classification further stratifies patients based on limitations in physical activity (5, 9). Together, these classifications provide essential tools for HF diagnosis and management.

Heart failure (HF) is a global health concern affecting approximately 26 million people, with prevalence rising significantly due to aging populations and improved survival rates for cardiovascular diseases. Around 10% of individuals over 70 years are affected, with lifetime risks estimated at 19.3% for HF with preserved ejection fraction (HFpEF) and 11.4% for HF with reduced ejection fraction (HFrEF) (11, 12). In Europe, HF prevalence is 1%–4%, with decompensated HF as the most common presentation, driven by coronary heart disease, hypertension, and atrial fibrillation. In Asia, prevalence ranges from 1%–3%, with younger patients presenting more severe symptoms compared to Western populations, primarily due to hypertension, diabetes, and ischemic heart disease, but with low adherence to guideline-directed therapies (13). Latin America reports an HF incidence of 199 cases per 100,000 person-years, a prevalence of 1%, an annual mortality rate of 24.5%, and an in-hospital mortality rate of 11.7%, particularly among patients with HFrEF, ischemic heart disease, and Chagas disease (14, 15). Ecuador shows an HF prevalence of 1%–2%, with poor prognosis linked to age, ethnicity, atrial fibrillation, and frequent hospitalizations (16). High-altitude settings, such as Quito, which is located at approximately 2,800 meters above sea level, provide a distinctive clinical context for cardiovascular care (17). Although altitude-related physiological adaptations have been described in cardiovascular research (18, 19), the clinical characterization of HF in Andean settings remains limited (20). In this context, describing the demographic, clinical, and hospitalization profile of patients treated in high-altitude hospitals may help address an important regional evidence gap, even when altitude itself is not directly evaluated as exposure.

A study by Crespo and Orellana reported a prevalence of 39.3 per 1,000 population from 2015 to 2019, primarily affecting older adults with comorbidities such as hypertension, arrhythmias, acute myocardial infarction, and type 2 diabetes mellitus (21, 22). Survival analysis in the Ecuadorian Andean population revealed one-year survival rates of 86% and five-year rates of 46%, with poor prognosis linked to factors including age, ethnicity, atrial fibrillation, frequent hospitalizations, and elevated creatinine levels.

Despite its increasing prevalence and burden, HF remains underexplored in Ecuador. This study aims to describe the clinical and epidemiological characteristics of patients diagnosed with HF at a tertiary private hospital in Quito between January 2021 and December 2023.

## 2 Methodology

### 2.1 Study design

A retrospective observational study was conducted to assess clinical outcomes and associated factors among patients diagnosed with heart failure. The study was based on the analysis of anonymized medical records from a tertiary hospital in Quito, Ecuador.

### 2.2 Setting

The study was conducted at the Hospital Metropolitano de Quito, a private specialty care center serving a predominantly private-sector population in Quito, Ecuador. Located at an altitude of approximately 2,800 meters above sea level, Quito is the capital of Ecuador and has a population of about 2.7 million (23).

### 2.3 Sample

Non-probabilistic consecutive sampling was employed to collect data from medical records, ensuring the inclusion of all eligible patients treated for heart failure during the study period. This approach enabled comprehensive case capture from the cardiology service between January 2021 and December 2023.

### 2.4 Inclusion and exclusion criteria

The inclusion criteria included patients aged ≥18 years with a confirmed diagnosis of heart failure based on the ICD-10 classification, including I500 (congestive heart failure, unspecified), I501 (acute systolic heart failure), and I509 (heart failure, unspecified). Eligible patients had received care at the Hospital Metropolitano de Quito between January 2021 and December 2023.

Exclusion criteria encompassed patients younger than 18 years, records lacking the minimum core clinical information required to confirm the diagnosis of heart failure and ascertain discharge outcome, records outside the study period, and diagnoses other than the specified ICD-10 codes.

### 2.5 Sample and data collection

**Figure 1:**
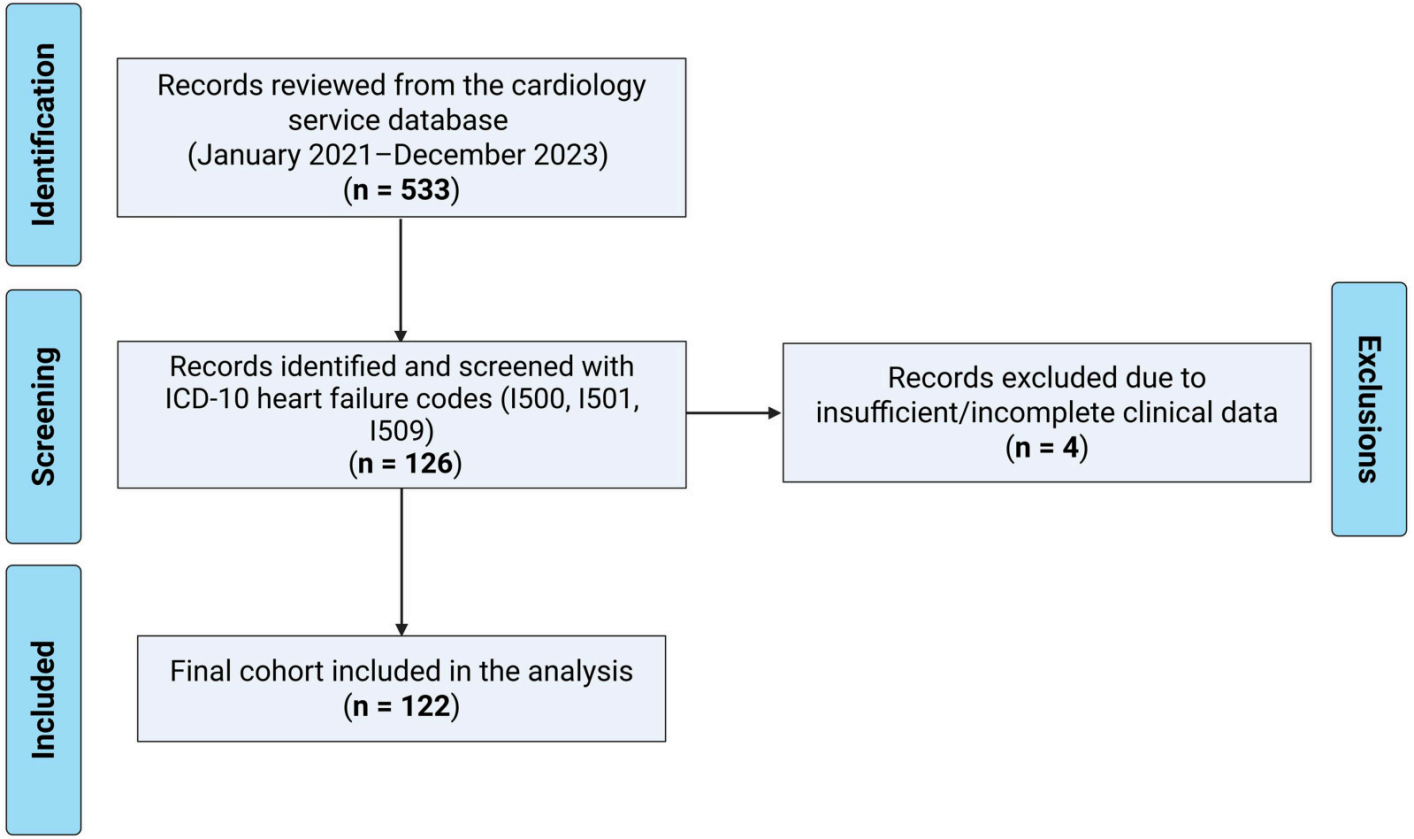
Flow diagram of record identification, exclusions, and final cohort included in the analysis (January 2021–December 2023).

From the cardiology service database (*n* = 533 total records reviewed during the study period), 126 clinical records corresponding to ICD-10 diagnoses of heart failure were identified. After applying the inclusion and exclusion criteria, 4 records were excluded because they lacked the minimum core information required for case confirmation or outcome ascertainment, resulting in a final sample size of 122 clinical records (Figure 1). Some eligible records still had missing values in specific secondary variables, particularly etiology and selected echocardiographic parameters, and these were retained in the descriptive analyses with variable-specific denominators. An anonymized database was created, which included demographic variables (e.g., sex, age at diagnosis, nationality, residence, ethnicity), anthropometric measurements, and clinical variables such as comorbidities, presentation forms, symptoms, types of heart failure, family history, treatment, and patient outcomes.

### 2.6 Statistical analysis

Descriptive statistics were used to summarize variables, with categorical data presented as frequencies and percentages and continuous variables reported as mean (standard deviation). Normality was assessed using the Shapiro–Wilk test, and continuous variables were approximately normally distributed in this dataset. Exploratory between-group comparisons were performed exclusively according to discharge survival status (alive vs. deceased). For categorical variables, chi-square tests were used (or Fisher's exact test when expected cell counts were small). For continuous variables, two-sample Student's *t*-tests were used to compare mean values between the two outcome groups. A two-sided *p* value <0.05 was considered statistically significant. All analyses were performed using IBM SPSS Statistics for Windows, version 29.0 (IBM Corporation, Chicago, IL, USA).

### 2.7 Ethical statement

The study adhered to the ethical principles outlined in the Declaration of Helsinki and was approved by the Ethics Committee for Research in Human Beings of the University of the Americas (CEISH-UDLA) under protocol code 2024-OBS-020. The research protocol, informed consent form, and database format were reviewed and approved. Given the retrospective and observational nature of the study, the ethics committee approved a waiver of informed consent, ensuring compliance with ethical and methodological standards. Patient confidentiality was rigorously protected through data anonymization, and all procedures conformed to the applicable ethical and legal regulations for research in Ecuador. Supporting documentation and evaluation forms are archived at CEISH-UDLA.

## 3 Results

### 3.1 Demographic and clinical characteristics of patients with heart failure

Among 122 patients with congestive heart failure, the majority were men (55.7%), aged over 65 years (88.5%), and of mixed ethnicity (97.5%). Only 3.3% (*n* = 4) of the patients died during the study period (Table 1).

**Table 1:** Clinical and demographic characteristics of the study population, stratified by survival Status.

Variable		Total		Death		Alive		*P*-value
		*n*	%	*n*	%	*n*	%	
Sex	Male	68	55.7	2	2.9	66	97.1	0.814
	Female	54	44.3	2	3.7	52	96.3	
Age Category (years)	<65	14	11.5	0	0.0	14	100.0	0.464
	>65	108	88.5	4	3.7	104	96.3	
Ethnicity	White	1	0.8	0	0	1	100	0.991
	Mestizo	119	97.5	4	3.4	115	96.6	
	Black	1	0.8	0	0	1	100	
	Other	1	0.8	0	0	1	100	
Hospitalization (30 Days)	No	110	90.2	4	3.6	106	96.4	0.502
	Yes	12	9.8	0	0.0	12	100.0	
Hospitalization (60 Days)	No	121	99.2	4	3.3	117	96.7	0.853
	Yes	1	0.8	0	0.0	1	100.0	
Smoking	No	93	76.2	4	4.3	89	95.7	0.256
	Yes	29	23.8	0	0.0	29	100.0	
Alcohol Consumption	No	115	94.3	4	3.5	111	96.5	0.616
	Yes	7	5.7	0	0.0	7	100.0	
History of Cardiac Surgery	No	103	84.4	3	2.9	100	97.1	0.597
	Yes	19	15.6	1	5.3	18	94.7	
Myocardial Infarction (MI)	No	102	83.6	3	2.9	99	97.1	0.636
	Yes	20	16.4	1	5.0	19	95.0	
Diabetes Mellitus Type 2 (DM2)	No	102	83.6	4	3.9	98	96.1	0.368
	Yes	20	16.4	0	0.0	20	100.0	
Hypertension (HTN)	No	49	40.2	1	2.0	48	98.0	0.529
	Yes	73	59.8	3	4.1	70	95.9	
Dyslipidemia	No	99	81.1	4	4.0	95	96.0	0.327
	Yes	23	18.9	0	0.0	23	100.0	
Other Vascular Diseases	No	110	90.2	2	1.8	108	98.2	**0**.**006**
	Yes	12	9.8	2	16.7	10	83.3	
Obesity	No	104	85.2	4	3.8	100	96.2	0.398
	Yes	18	14.8	0	0.0	18	100.0	
Chronic Kidney Disease (CKD)	No	105	86.1	3	2.9	102	97.1	0.516
	Yes	17	13.9	1	5.9	16	94.1	
Dialysis	None	118	96.7	3	2.5	115	97.5	**0**.**013**
	Yes	4	3.3	1	25.0	3	75.0	
Immunologic Disease	No	114	93.4	3	2.6	111	97.4	**<0**.**001**
	Yes	8	6.6	1	12.5	7	87.5	

Categorical variables are presented as *n* (%). *P* values compare patients who died vs. those alive (chi-square or Fisher's exact tests, as appropriate).

Statistically significant result.

Comorbid conditions included arterial hypertension (59.8%) as the most prevalent, followed by dyslipidemia (18.9%). Additionally, 16.4% had experienced a myocardial infarction, and 15.6% had a history of cardiac surgery (Table 1).

### 3.2 Etiology and hospitalization details

**Figure 2:**
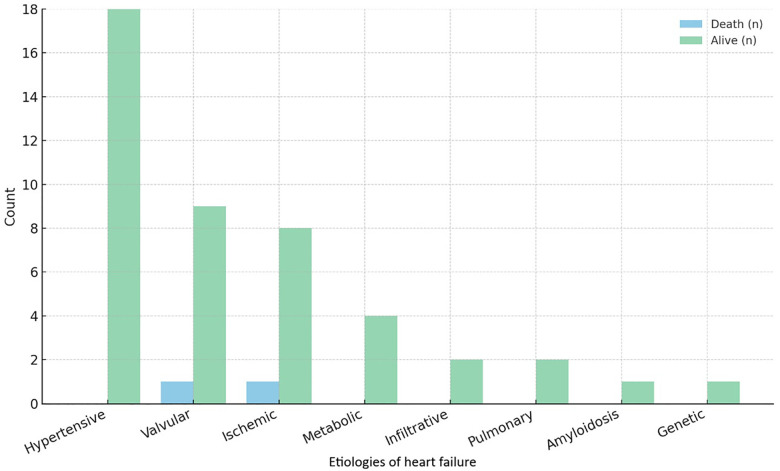
Documented etiologies of heart failure in the cohort by patient outcome. Analyses were restricted to records with a documented etiology (*n* = 46); etiology was not available in 76 records.

Hypertensive heart disease was the most frequent documented etiology of heart failure, accounting for 14.8% (*n* = 18) of the full cohort and 39.1% of cases with available etiologic information (Figure 2). At admission, 87.7% of patients presented in an unstable condition. The mean heart rate was 82.0 ± 21.3 bpm, with a mean systolic blood pressure of 120.1 ± 28.4 mmHg and a diastolic blood pressure of 71.0 ± 12.9 mmHg. While 29.5% of patients were admitted primarily for heart failure, a larger proportion (71.3%) exhibited heart failure symptoms, with dyspnea (63.1%) being the most frequent, followed by edema (45.9%). Atrial fibrillation was identified in 44.3% of cases, while acute coronary syndrome was reported in only 3.3% (Table 2).

**Table 2:** Admission Status, cardiac conditions, and comorbidities of the study population stratified by survival Status.

Variable		Total		Death		Alive		*P*-Value
		*n*	%	*n*	%	*n*	%	
Admission Status	Stable	11	9	0	0.0	11	100.0	0.748
	Unstable	107	87.7	4	3.7	103	96.3	
Heart Rate (bpm)	Mean (*±SD*)	82.0	21.3	110.0	32.8	81.1	20.4	0.007*
Systolic Blood Pressure (mmHg)	Mean (*±SD*)	120.1	28.4	104.0	24.0	120.7	28.4	0.249*
Diastolic Blood Pressure (mmHg)	Mean (*±SD*)	71.0	12.9	74.0	21.8	70.9	12.6	0.637*
Hemoglobin (g/dL)	Mean (*±SD*)	13.1	2.6	11.3	1.9	13.1	2.6	0.067*
Hematocrit (%)	Mean (*±SD*)	39.2	8.0	34.6	5.7	39.4	8.0	0.098*
Creatinine (mg/dL)	Mean (*±SD*)	1.8	4.2	2.0	1.7	1.7	4.3	0.779*
Troponin (ng/L)	Mean (*±SD*)	74.3	146.6	341.2	473.0	59.7	95.9	0.320*
ProBNP (pg/mL)	Mean (*±SD*)	5,994.6	7,185.0	15,088.2	12,623.6	5,500.4	6,531.8	0.165*
Heart Failure (HF)	No	86	70.5	0	0	86	100	0.009
	Yes	36	29.5	4	3.3	32	96.7	
Atrial Fibrillation	No	68	55.7	2	2.9	66	97.1	0.822
	Yes	54	44.3	2	3.7	52	96.3	
Arrhythmia (other than AF)	No	111	91	3	2.7	108	97.3	0.256
	Yes	11	9	1	9.1	10	90.9	
Type of Arrhythmia	None	115	94.3	3	2.6	112	97.4	0.184
	Block	1	0.8	0	0.0	1	100.0	
	Unknown	4	3.3	1	25.0	3	75.0	
	Ventricular Ectopy	1	0.8	0	0.0	1	100.0	
	Persistent	1	0.8	0	0.0	1	100.0	
Pacemaker use	No	108	88.5	3	2.8	105	97.2	0.388
	Yes	14	11.5	1	7.1	13	92.9	
Acute Coronary Syndrome (ACS)	No	118	96.7	3	2.5	115	97.5	0.814
	Yes	4	3.3	1	25.0	3	75.0	
Heart Failure Symptoms	No	35	28.7	0	0.0	35	100.0	0.197
	Yes	87	71.3	4	4.6	83	95.4	
Dyspnea	No	45	36.9	1	2.2	44	97.8	0.616
	Yes	77	63.1	3	3.9	74	96.1	
Edema	No	66	54.1	2	3.0	64	97.0	0.867
	Yes	56	45.9	2	3.6	54	96.4	
Palpitations	No	110	90.2	4	3.6	106	96.4	0.502
	Yes	12	9.8	0	0.0	12	100.0	
Angina	No	111	91.0	4	3.6	107	96.4	0.522
	Yes	11	9.0	0	0.0	11	100.0	

Categorical variables are presented as *n* (%), and continuous variables as mean (SD). *P* values compare patients who died vs. those alive. For categorical variables, *p* values were obtained using chi-square or Fisher's exact tests (as appropriate). For continuous variables (marked with *), *p* values were obtained using two-sample Student's *t*-tests.

### 3.3 Patient characteristics at discharge

At discharge, most echocardiographic parameters—including left ventricular ejection fraction (LVEF), E/e' ratio, right ventricular systolic pressure (RVSP), and left atrial volume index (LAVI)—did not show significant associations with in-hospital mortality. However, the presence of akinesia as a wall motion abnormality was significantly associated with increased mortality (28.6% vs. 2.1% in those without, *p* = 0.001), while hypokinesia was not. In terms of pharmacological therapy, the absence of beta-blocker prescription was associated with higher mortality (7.1% vs. 0% in those receiving beta-blockers, *p* = 0.027). Similarly, the absence of a mineralocorticoid receptor antagonist was associated with greater mortality (7.5% vs. 0% among those prescribed an MRA, *p* = 0.020) (Table 3).

**Table 3:** Echocardiographic characteristics and pharmacological therapy at discharge and their association with in-hospital mortality.

		Total		Death		Alive		*p* value
		*n*	%	*n*	%	*n*	%	
Echocardiographic Variables								
Left Ventricular Ejection Fraction (LVEF)	Preserved	59	48.4	1	1.7	58	98.3	0.286
	Mildly reduced	19	15.6	0	0.0	19	100.0	
	Reduced	9	7.4	1	11.1	8	88.9	
	Severely reduced	8	6.6	1	12.5	7	87.5	
	NA	27	22.1	1	3.7	26	96.3	
E/e’ ratio^a^	Normal	10	8.2	0	0.0	10	100.0	0.423
	Gray zone	37	30.3	0	0.0	37	100.0	
	Elevated	31	25.4	2	6.5	29	93.50	
	NA	44	36.1	2	4.5	42	95.50	
Right Ventricular TAPSE	Normal	52	42.6	0	0.0	52	100.0	0.215
	Reduced	36	29.5	2	5.6	34	94.4	
	NA	34	27.9	2	5.9	32	94.1	
Right Ventricular Systolic Pressure (RVSP)	Normal	14	11.5	0	0.0	14	100.0	0.602
	Borderline	15	12.3	0	0.0	15	100.0	
	Elevated	29	23.8	1	3.4	28	96.6	
	Severely elevated	15	12.3	0	0.0	15	100.0	
	NA	49	40.2	3	6.1	46	93.9	
Left Atrial Volume Index (LAVI)	Normal	14	11.5	1	7.1	13	92.9	0.755
	Mild dilation	9	7.4	0	0.0	9	100.0	
	Moderate	13	10.7	0	0.0	13	100.0	
	Severe	45	36.9	1	2.2	44	97.8	
	NA	41	33.6	2	4.9	39	95.1	
Wall Motion Abnormalities	No	101	82.8	2	2.0	99	98.0	0.077
	Yes	21	17.2	2	9.5	19	90.5	
Type of Wall Motion Abnormality	Akinesia	7	5.7	2	28.6	5	71.4	0.001
	Hypokinesia	21	17.2	0	0.0	21	100.0	
	NA	94	77.0	2	2.1	92	97.9	
Location of Wall Motion Abnormality	Anterior	14	11.5	1	7.1	13	92.9	0.288
	Apical	4	3.3	1	25.0	3	75.0	
	Global	4	3.3	0	0.0	4	100.0	
	Inferior	5	4.1	0	0.0	5	100.0	
	Lateral	2	1.6	0	0.0	2	100.0	
	Septal	1	0.8	0	0.0	1	100.0	
	No	92	75.4	2	2.2	90	97.8	
Pharmacological Therapy at Discharge								
Beta-blocker	No	56	45.9	4	7.1	52	92.9	0.027
	Yes	66	54.1	0	0.0	66	100.0	
ACE Inhibitor (ACEi)	No	98	80.3	4	4.1	94	95.9	0.314
	Yes	24	19.7	0	0.0	24	100.0	
Statin	No	92	75.4	3	3.3	89	96.7	0.985
	Yes	30	24.6	1	3.3	29	96.7	
Antiplatelet Therapy	No	105	86.1	3	2.9	102	97.1	0.516
	Yes	17	13.9	1	5.9	16	94.1	
Anticoagulation	No	49	40.2	3	6.1	46	93.9	0.148
	Yes	73	59.8	1	1.4	72	98.6	
Type of Anticoagulant	Apixaban	47	38.5	0	0.0	47	100.0	0.451
	Dabigatran	5	4,10	0	0,0	5	100,0	
	Enoxaparin	8	6,60	0	0,0	8	100,0	
	Rivaroxaban	13	10,70	1	7,7	12	92,3	
	Warfarin	10	8,20	0	0,0	10	100,0	
	Other	1	0,80	0	0,0	1	100,0	
	No	38	31,10	3	7,9	35	92,1	
Mineralocorticoid receptor antagonist	No	53	56.6	4	7.5	49	92.5	**0**.**020**
	Yes	69	43.4	0	0.0	69	100.0	
Neprilysin inhibitors (NEPIs)	No	106	86.9	4	3.8	102	96.2	0.429
	Yes	16	13.1	0	0.0	16	100.0	
SGLT2 inhibitor	No	87	71.3	4	4.6	83	95.4	0.197
	Yes	35	28.7	0	0.0	35	100.0	
Functional Class at Discharge								
NYHA class	I	9	7,40	1	11,1	8	88,9	0,449
	II	40	32,80	0	0,0	40	100,0	
	III	17	13,90	1	5,9	16	94,1	
	IV	5	4,10	0	0,0	5	100,0	
	NA	51	41,80	2	3,9	49	96,1	

a The grouping parameters for the “E/e′ ratio” variable were defined based on the cutoff values established in the 2016 ASE/EACVI guidelines and the review by Zamfirescu. et al. (24, 25). Variables are presented as *n* (%). *P* values compare patients who died vs. those alive (chi-square or Fisher's exact tests, as appropriate).

Statistically significant result.

**Figure 3:**
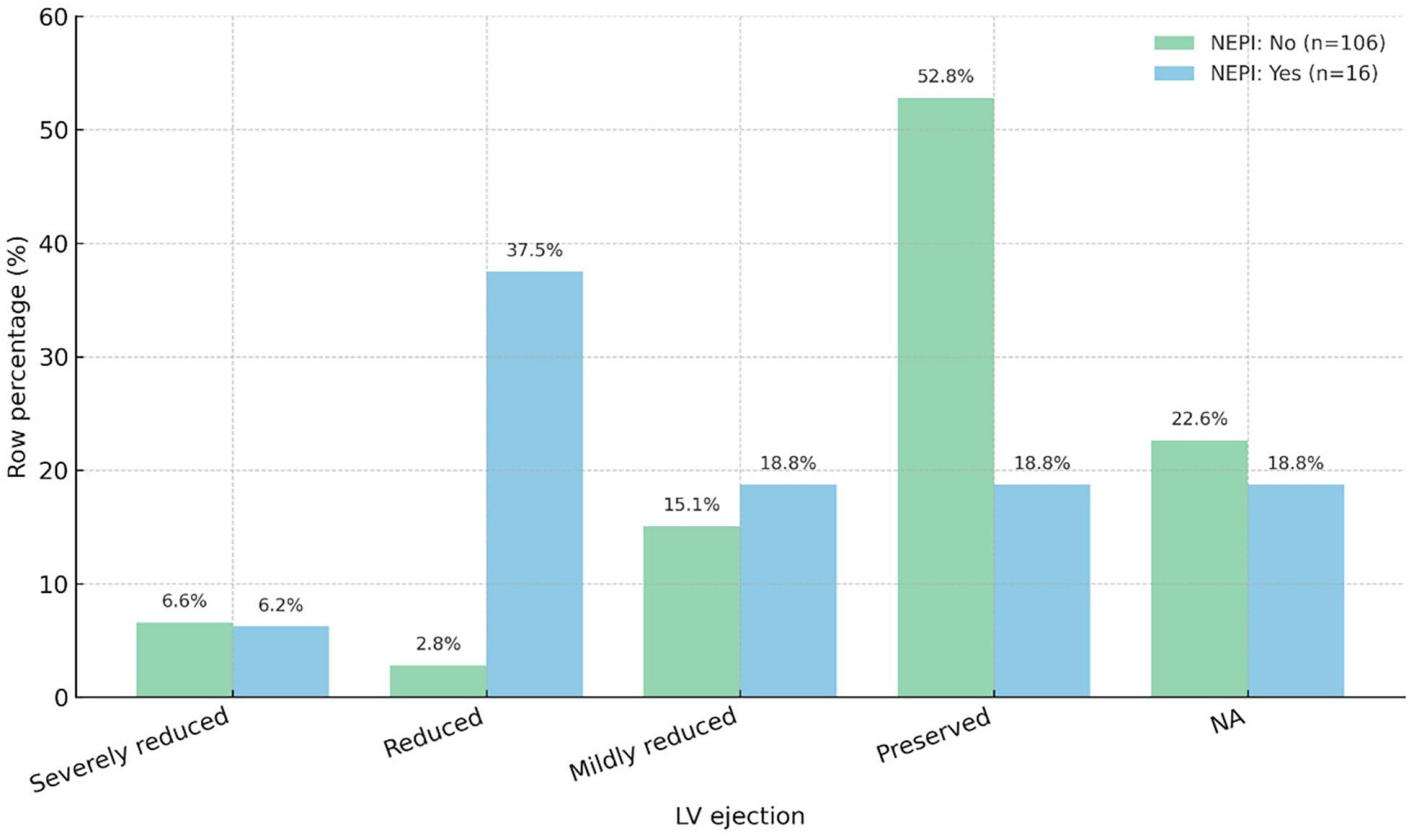
Distribution of qualitative LV ejection fraction categories by neprilysin inhibitor (sacubitril/valsartan) use at discharge. This figure summarizes treatment patterns in the cohort and illustrates that NEPI use clustered in lower LVEF categories, consistent with guideline-directed treatment selection in HFrEF.

Among patients without neprilysin inhibitors (NEPI; *n* = 106), over half had preserved LV ejection (52.8%), with smaller proportions mildly reduced (15.1%); in contrast, the NEPI use group (*n* = 16) showed a shift toward impairment—reduced 37.5% and preserved 18.8% (mildly reduced 18.8%, severely reduced 6.2%), *p* < 0.001, indicating NEPI use clustered in lower LVEF categories, consistent with treatment selection in HFrEF (Figure 3).

### 3.4 Characteristics of deceased patients

Heart failure-related mortality analysis revealed that among the four deceased patients, 50% were male, and 100% were of mestizo ethnicity. Hypertension was present in all cases (100%) as a preexisting condition. During hospitalization, the patients had a mean systolic blood pressure of 104.0 ± 24.0 mmHg and a diastolic blood pressure of 74.0 ± 21.8 mmHg. Atrial fibrillation was observed in 50% of cases, while dyspnea (75.0%) and edema (50.0%) were the most frequently reported symptoms (Supplementary Table S1).

### 3.5 Exploratory unadjusted associations with in-hospital mortality

Demographic variables, including sex, age, and ethnicity, showed no significant differences between survivors and non-survivors. However, a higher prevalence of mortality was observed among patients with other vascular diseases (16.7%, *p* = 0.006). Although chronic kidney disease alone was not significantly associated with mortality, patients undergoing dialysis exhibited a markedly higher death rate (25%, *p* = 0.013). Additionally, immunological diseases were significantly more frequent among deceased patients (12.5%, *p* < 0.001) (Table 1).

In terms of hospitalization characteristics, admission status, arrhythmias, acute coronary syndrome, and pacemaker use were similarly distributed between survivors and non-survivors. However, the heart rate was significantly higher among deceased patients, with a mean of 110.0 ± 32.8 bpm (*p* = 0.007). Furthermore, all patients who died belonged to the group recorded as having heart failure at admission (*p* = 0.009); however, given the very small number of events, this finding should be interpreted cautiously (Table 2).

## 4 Discussion

This study provides a descriptive analysis of the demographic and clinical characteristics of patients with HF treated in a high-altitude city and explores unadjusted associations with in-hospital mortality. As one of the few studies conducted in Ecuador, this research contributes significantly to understanding HF within a unique environmental and demographic context. The findings largely align with existing global literature, but they also underscore the relevance of describing HF in a high-altitude care setting. Because this retrospective design lacks standardized hypoxia-related measures and does not include a sea-level comparator cohort, altitude-specific inferences should be interpreted cautiously.

High-altitude living is associated with reduced oxygen availability and physiological adaptations that may affect cardiovascular function (20, 26). However, this study was not designed to phenotype hypoxia adaptation or maladaptation, nor to isolate altitude as an exposure, because standardized hypoxia-related measures (e.g., oxygen saturation/arterial blood gases, duration of residence at altitude, validated chronic mountain sickness assessments, or systematic pulmonary hemodynamic measures) were not consistently available in this retrospective dataset and we did not include a sea-level comparator cohort (27).

We observed a high prevalence of atrial fibrillation (44.3%) in our cohort. Although altitude-related physiology has been proposed as a potential contributor to arrhythmic risk in some populations (28), our retrospective design and lack of standardized hypoxia-related measures preclude causal inferences regarding altitude-driven mechanisms (29). Therefore, this finding should be interpreted as a descriptive characteristic of this hospital-based cohort.

Our results demonstrated that HF predominantly affects older adults, with 88.5% of patients aged over 65 years, and is more common among men (55.7%). These findings align with global data indicating that HF is most prevalent in older adults and highlights the importance of addressing aging-related cardiovascular risks (30). However, the relatively high proportion of male patients contrasts with reports from other regions, which suggest an increasing burden of HF among women and highlights the need to investigate potential sex-specific risk factors in Ecuadorian populations (31).

ypertension, observed in 59.8% of patients, was the most common comorbidity, reinforcing its well-documented role as a major risk factor for HF (32). Although altitude-related physiology has been proposed as a possible modifier of blood pressure regulation in some settings (20), our study was not designed to evaluate this mechanism directly. The study also identified dyslipidemia and prior myocardial infarction as significant contributors to HF pathogenesis, consistent with global findings.

The study's in-hospital mortality rate was low (3.3%), and the mortality-related analyses should therefore be interpreted as exploratory. In unadjusted comparisons, dialysis dependency, immunologic diseases, and other vascular diseases showed associations with in-hospital mortality; however, these findings are based on a very small number of events and should not be interpreted as independent predictors (33). These findings align with previous studies showing that vascular and renal comorbidities exacerbate HF progression (34).

The high-altitude clinical setting in which this study was conducted provides relevant descriptive context for HF care in Quito. Although previous literature suggests that altitude exposure may influence cardiopulmonary physiology in some patients with HF (29, 35), these mechanisms were not directly evaluated in our study and should not be inferred from our findings.

In addition, the predominance of mixed ethnicity in this study population suggests the need to consider genetic and environmental factors that may influence HF presentation and outcomes in Ecuador. Although chronic mountain sickness has been described in high-altitude populations and may be associated with distinct cardiopulmonary remodeling (27), we did not systematically assess or diagnose chronic mountain sickness in this retrospective cohort; therefore, we cannot evaluate its contribution to HF phenotypes or outcomes in our study. Future studies should investigate the interplay between genetic predispositions, environmental factors, and altitude-related clinical contexts in order to better characterize HF phenotypes in Andean populations. Additional multicenter studies with standardized physiological measurements and appropriate comparator cohorts will be necessary before altitude-specific management approaches or regionally tailored recommendations can be considered.

## 5 Strengths and limitations

The main strength of this study lies in describing the clinical and epidemiological profile of patients with HF treated in a high-altitude hospital setting, an area that remains underreported in Ecuador and the Andean region. The study contributes contextual hospital-based data from Quito, although it was not designed to evaluate altitude-related mechanisms or their impact on HF outcomes.

However, the study is limited by its relatively small sample size and single-center design, which may not fully capture the diversity of HF presentations across Ecuador. The retrospective nature of the data collection may have introduced selection bias and limited the ability to establish causal relationships between variables. Missing or incomplete clinical information may have also affected the accuracy of the results. Additionally, the predominance of patients of mixed ethnicity in this study further limits the applicability of the findings to other ethnic groups with different risk factors or outcomes. Unmeasured confounding factors, such as socioeconomic status or lifestyle habits, could have influenced the observed associations.

Importantly, this retrospective chart review did not include standardized measures needed to phenotype hypoxia adaptation or maladaptation (e.g., oxygen saturation/arterial blood gases, duration of residence at altitude, validated chronic mountain sickness assessments, or systematic pulmonary hemodynamic measures). In addition, no sea-level comparator cohort was available; therefore, altitude was not evaluated as an exposure or risk factor and altitude-specific mechanisms cannot be inferred. Consequently, the findings should be interpreted primarily as a descriptive characterization of HF patients treated in a high-altitude clinical setting. Additionally, the statistical analysis was limited to univariate comparisons; given the low number of events, multivariable modeling was not feasible, and therefore the observed associations should not be interpreted as independent predictors. On the other, the low number of in-hospital deaths (*n* = 4) substantially limits the statistical power and stability of comparisons between survivors and non-survivors. Consequently, findings related to mortality should be interpreted with caution and considered exploratory. Finally, several key clinical variables were missing or incompletely documented in a substantial proportion of patients. For example, HF etiology was unavailable in 76 of 122 records, while several echocardiographic parameters also showed missing data, including E/e' ratio (44/122), RVSP (49/122), LAVI (41/122), and TAPSE (34/122). This incomplete documentation may have affected the accuracy, interpretation, and generalizability of the findings. Thus, the exclusion of records with insufficient core data should not be interpreted as implying complete data availability across all study variables; rather, some included records had partial missingness in secondary clinical and echocardiographic fields.

## 6 Conclusion

This study provides a contemporary clinical and epidemiological characterization of HF patients treated at a tertiary hospital in Quito (−2,800 m a.s.l.) and describes exploratory, unadjusted associations with in-hospital mortality that should be interpreted cautiously given the very low number of events. Overall, the study should be understood as a descriptive analysis of HF patients managed in a high-altitude clinical setting rather than as an evaluation of altitude as an independent determinant of outcome. These findings support the need for future studies incorporating standardized hypoxia-related measurements and appropriate comparator cohorts to better understand HF phenotypes and outcomes in Andean populations.

These descriptive findings may help inform the design of future multicenter studies focused on heart failure in Andean and other high-altitude settings. Further research incorporating standardized hypoxia-related measurements, more complete clinical phenotyping, and appropriate comparator cohorts will be necessary before altitude-specific management strategies or regionally tailored recommendations can be considered.

## Data Availability

The original contributions presented in the study are included in the article/Supplementary Material, further inquiries can be directed to the corresponding author/s.

## References

[1] John EH Michael EH. Guyton and Hall Textbook of Medical Physiology. Canada: Elsevier (2020).

[2] Rao A Gupta A Kain V Halade GV. Extrinsic and intrinsic modulators of inflammation-resolution signaling in heart failure. Am J Physiol Heart Circ Physiol. (2023) 325(3):H433–48. 10.1152/ajpheart.00276.2023 37417877 PMC10538986

[3] Rosenkranz S Gibbs JSR Wachter R De Marco T Vonk-Noordegraaf A Vachiéry JL. Left ventricular heart failure and pulmonary hypertension. Eur Heart J. (2016) 37(12):942–54. 10.1093/eurheartj/ehv512 26508169 PMC4800173

[4] Golla MSG Hajouli S Ludhwani D. Heart Failure and Ejection Fraction. En: StatPearls. Treasure Island (FL): StatPearls Publishing (2025). 31971755

[5] Inamdar AA Inamdar AC. Heart failure: diagnosis, management and utilization. J Clin Med. (2016) 5(7):62. 10.3390/jcm5070062 27367736 PMC4961993

[6] Sengupta PP Narula J. Reclassifying heart failure: predominantly subendocardial, subepicardial, and transmural. Heart Fail Clin Julio de. (2008) 4(3):379–82. 10.1016/j.hfc.2008.03.013 18598989

[7] National Institutes of Health. Heart Failure - What Is Heart Failure? (2022). Available online at: https://www.nhlbi.nih.gov/health/heart-failure [citado 22 de febrero de 2025] (Accessed January 7, 2025).

[8] Sachdev V Sharma K Keteyian SJ Alcain CF Desvigne-Nickens P Fleg JL Supervised exercise training for chronic heart failure with preserved ejection fraction: a scientific statement from the American Heart Association and American College of Cardiology. Circulation. (2023) 147(16):e699–715. 10.1161/CIR.0000000000001122 36943925 PMC12019885

[9] American Heart Association. www.heart.org. Classes and stages of heart failure (2023). Available online at: https://www.heart.org/en/health-topics/heart-failure/what-is-heart-failure/classes-of-heart-failure [citado 22 de febrero de 2025] (Accessed January 7, 2025).

[10] Redfield MM Borlaug BA. Heart failure with preserved ejection fraction: a review. JAMA. (2023) 329(10):827–38. 10.1001/jama.2023.2020 36917048

[11] Institute for Quality and Efficiency in Health Care (IQWiG). Overview: Heart Failure. En: InformedHealth.org. 1st ed. Institute for Quality and Efficiency in Health Care (IQWiG) (2023). Available online at: https://www.ncbi.nlm.nih.gov/books/NBK279539/ [citado 22 de febrero de 2025]

[12] Ponikowski P Anker SD AlHabib KF Cowie MR Force TL Hu S Heart failure: preventing disease and death worldwide. ESC Heart Fail. (2014) 1(1):4–25. 10.1002/ehf2.12005 28834669

[13] Yoo SGK Ahmed MO Sweitzer NK. Current and future of heart failure care in Asia. Int J Heart Fail. (2024) 6(4):141–8. 10.36628/ijhf.2024.0033 39513020 PMC11538722

[14] Ciapponi A Alcaraz A Calderón M Matta M. Carga de enfermedad de la insuficiencia cardiaca en américa Latina: revisión sistemática y metanálisis. Rev Esp Cardiol. (2016) 69(11):1051–60. 10.1016/j.recesp.2016.04.045 27553287

[15] Vásconez-González J Izquierdo-Condoy JS Fernandez-Naranjo R Gamez-Rivera E Tello-De-la-Torre A Guerrero-Castillo GS Severe chagas disease in Ecuador: a countrywide geodemographic epidemiological analysis from 2011 to 2021. Front Public Health. (2023) 11:1172955. 10.3389/fpubh.2023.1172955 37143984 PMC10151800

[16] Moreno-Rondón L Ortega-Armas ME Coronel A Vaca I Guevara B Alarcón Cedeño R Characteristics, treatment and prognosis of patients with chronic heart failure according to ejection fraction. Results of an Ecuadorian registry. Acta Cardiol. (2024) 79(8):942–52. 10.1080/00015385.2024.2392335 39161326

[17] Ortiz-Prado E Izquierdo-Condoy JS Fernández-Naranjo R Vásconez-González J Cano L González AC Epidemiological characterization of ischemic heart disease at different altitudes: a nationwide population-based analysis from 2011 to 2021 in Ecuador. PLoS One. (2023) 18(12):e0295586. 10.1371/journal.pone.0295586 38157383 PMC10756509

[18] Ortiz-Prado E Dunn JF Vasconez J Castillo D Viscor G. Partial pressure of oxygen in the human body: a general review. Am J Blood Res. (2019) 9(1):1–14. 30899601 PMC6420699

[19] Izquierdo-Condoy JS Arias-Rodríguez FD Díaz-Chamba WI Mena-Noroña DA Toaquiza LC Espín-Sambache B Epidemiological characterization of congenital heart disease at different altitudes in Ecuador: a four-year retrospective study in a pediatric referral hospital. Front Public Health. (2025) 13:1497253. 10.3389/fpubh.2025.1497253 39944066 PMC11813749

[20] Huez S Faoro V Vachiéry JL Unger P Martinot JB Naeije R. Images in cardiovascular medicine. High-altitude-induced right-heart failure. Circulation. (2007) 115(9):e308–309. 10.1161/CIRCULATIONAHA.106.650994 17339556

[21] Sanchez JAF Vera JJM Lopera KLS Medranda EFC. Factors influencing heart failure in adult patients. Univ Cienc Tecnol. (2023) 27(119):116–23. 10.47460/uct.v27i119.712

[22] Crespo APC Orellana KPO. Prevalencia y factores asociados a insuficiencia cardiaca en adultos mayores. Hospital homero castanier crespo, 2015–2019. Rev Fac Cienc Médicas Univ Cuenca. (2021) 39(2):11–9. 10.18537/RFCM.39.02.03

[23] Distrito Metropolitano de Quito. Quito Cómo Vamos. Explora los datos – Demografía (2023). Available online at: https://quitocomovamos.org/explora-los-datos/ [citado 22 de noviembre de 2023] (Accessed November 21, 2023).

[24] Nagueh SF Smiseth OA Appleton CP Byrd BF Dokainish H Edvardsen T Recommendations for the evaluation of left ventricular diastolic function by echocardiography: an update from the American society of echocardiography and the European association of cardiovascular imaging. J Am Soc Echocardiogr Off Publ Am Soc Echocardiogr. (2016) 29(4):277–314. 10.1016/j.echo.2016.01.011 27037982

[25] Zamfirescu MB Ghilencea LN Popescu MR Bejan GC Maher SM Popescu AC The E/e’ ratio—role in risk stratification of acute heart failure with preserved ejection fraction. Medicina (Mex). (2021) 57(4):375. 10.3390/medicina57040375 PMC8070491 33924367

[26] Mallet RT Burtscher J Richalet JP Millet GP Burtscher M. Impact of high altitude on cardiovascular health: current perspectives. Vasc Health Risk Manag. (2021) 17:317–35. 10.2147/VHRM.S294121 34135590 PMC8197622

[27] Pena E El Alam S Siques P Brito J. Oxidative stress and diseases associated with high-altitude exposure. Antioxid Basel Switz. (2022) 11(2):267. 10.3390/antiox11020267 PMC8868315 35204150

[28] Higgins JP Tuttle T Higgins JA. Altitude and the heart: is going high safe for your cardiac patient? Am Heart J. Enero de. (2010) 159(1):25–32. 10.1016/j.ahj.2009.10.028 20102863

[29] Doutreleau S Ulliel-Roche M Hancco I Bailly S Oberholzer L Robach P Cardiac remodelling in the highest city in the world: effects of altitude and chronic mountain sickness. Eur J Prev Cardiol. (2022) 29(17):2154–62. 10.1093/eurjpc/zwac166 35929776

[30] Agostoni P. Considerations on safety and treatment of patients with chronic heart failure at high altitude. High Alt Med Biol. (2013) 14(2):96–100. 10.1089/ham.2012.1117 23795728

[31] Kaya A Bayramoğlu A Bektaş O Yaman M Günaydın ZY Topcu S The prognostic value of altitude in patients with heart failure with reduced ejection fraction. Anatol J Cardiol. (2019) 22(6):300–8. 10.14744/anatoljcardiol.2019.81535 31789616 PMC6955054

[32] Blue B. Journeys to high altitude–risks and recommendations for travelers with preexisting medical conditions. J Travel Med. (2010) 17(3):214. 10.1111/j.1708-8305.2010.00414.x 20536897

[33] Mikołajczak K Czerwińska K Pilecki W Poręba R Gać P Poręba M. The impact of temporary stay at high altitude on the circulatory system. J Clin Med. (2021) 10(8):1622. 10.3390/jcm10081622 33921196 PMC8068881

[34] Letourneau MM Brancheau D Estes J Zughaib M. Take me higher: a case of heart failure at high altitude detected using the CardioMEMS^TM^ HF system. Am J Case Rep. (2020) 21:e922857-1–857-5. 10.12659/AJCR.922857 32966270 PMC7520869

[35] Schmid JP Nobel D Brugger N Novak J Palau P Trepp A Short-term high altitude exposure at 3454 m is well tolerated in patients with stable heart failure. Eur J Heart Fail. (2015) 17(2):182–6. 10.1002/ejhf.227 25597947

