# Supplementary material for: Clinical and epidemiological characterization of heart failure patients in a high-altitude setting: a retrospective study at a tertiary hospital in Quito, Ecuador
Source: Front Cardiovasc Med. 2026 Apr 21;13:1776502. doi: 10.3389/fcvm.2026.1776502 (PMC13139094; doi:10.3389/fcvm.2026.1776502)
Supplement: Supplementary file 1 [file Table1.docx]

**Supplementary Table 1.** Characteristics of deceased patients diagnosed with heart failure.

|  |  | **n** | **%** |
| --- | --- | --- | --- |
| Sex | Male | 2 | 50.0 |
|  | Female | 2 | 50.0 |
| Ethnicity | Mestizo | 4 | 100.0 |
| Hospitalization (30 Days) | No | 4 | 100.0 |
| Hospitalization (60 Days) | No | 4 | 100.0 |
| Smoking | No | 4 | 100.0 |
| Alcohol Consumption | No | 4 | 100.0 |
| History of Cardiac Surgery | No | 3 | 75.0 |
|  | Yes | 1 | 25.0 |
| Myocardial Infarction (MI) | No | 3 | 75.0 |
|  | Yes | 1 | 25.0 |
| Type of MI | NSTEMI | 1 | 25.0 |
|  | None | 3 | 75.0 |
| Hypertension (HTN) | No | 1 | 25.0 |
|  | Yes | 3 | 75.0 |
| Diabetes Mellitus Type 2 (DM2) | No | 4 | 100.0 |
| Dyslipidemia | No | 4 | 100.0 |
| Other Vascular Diseases | No | 2 | 50.0 |
|  | Yes | 2 | 50.0 |
| Obesity | No | 4 | 100.0 |
| Chronic Kidney Disease (CKD) | No | 3 | 75.0 |
|  | Yes | 1 | 25.0 |
| **Admission and hospitalization** |  |  |  |
| Admission Status | Stable | 0 | 0.00 |
|  | Unstable | 4 | 100.0 |
| Heart Rate (bpm) | Mean (±SD) | 110.0 | 32.8 |
| Systolic Blood Pressure (mmHg) | Mean (±SD) | 104.0 | 24.0 |
| Diastolic Blood Pressure (mmHg) | Mean (±SD) | 74.0 | 21.8 |
| Heart Failure | Yes | 4 | 100.0 |
| Pacemaker use | No | 3 | 75.0 |
|  | Yes | 1 | 25.0 |
| Atrial Fibrillation | No | 2 | 50.0 |
|  | Yes | 2 | 50.0 |
| Acute Coronary Syndrome | No | 3 | 75.0 |
|  | Yes | 1 | 25.0 |
| **Heart failure symptoms** |  |  |  |
| Dyspnea | No | 1 | 25.0 |
|  | Yes | 3 | 75.0 |
| Palpitations | No | 4 | 100.0 |
| Edema | No | 2 | 50.0 |
|  | Yes | 2 | 50.0 |
| Angina | No | 4 | 100.0 |
